# Inquiry into the short- and long-term effects of Roux-en-Y gastric bypass on the glomerular filtration rate

**DOI:** 10.1080/0886022X.2020.1790389

**Published:** 2020-07-13

**Authors:** Allon N. Friedman, Robert V. Considine, Sara K. Quinney

**Affiliations:** a Department of Medicine, Indiana University School of Medicine, Indianapolis, IN, USA; b Department of Obstetrics and Gynecology, Indiana University School of Medicine, Indianapolis, IN, USA

**Keywords:** Bariatric surgery, Roux-en Y gastric bypass, glomerular filtration rate, GFR, GLP-1, glucagon like-peptide 1, obesity, weight

## Abstract

Bariatric surgery is known to attenuate glomerular hyperfiltration over the long term and thereby protect the kidney from mechanical damage. Whether this effect is directly related to weight loss or is independent of weight as are some of its other beneficial metabolic effects is not known. We explored this question in a preliminary study that directly measured glomerular filtration rate (GFR) before, immediately after, and again many months after Roux-en-Y gastric bypass after large weight loss had occurred. We simultaneously measured stimulated circulating glucagon-like peptide-1, which is upregulated after Roux-en-Y gastric bypass and is a putative mediator of GFR after bariatric surgery. We found no weight-independent effect of Roux-en-Y gastric bypass on GFR nor an association between circulating GLP-1 levels and GFR. These findings, if confirmed in larger studies, will help steer future enquiries in this area.

## Introduction

Obesity is arguably the most important modifiable cause of CKD in the modern era because it is prevalent in nearly one in two patients with CKD and promotes kidney damage through direct effects and intermediate risk factors like diabetes and hypertension. Among its most important direct adverse effects is glomerular hyperfiltration, which can lead to maladaptive changes that damage the glomerulus and proximal tubule [1]. Attenuation of obesity-related glomerular hyperfiltration is therefore an important strategy for renoprotection.

Of all available weight reduction interventions bariatric surgery offers the most effective and sustained results. Bariatric surgery is also known to reverse glomerular hyperfiltration when measured months to years post-operatively [2]. This has led to the belief that attenuation of glomerular hyperfiltration is a direct result of weight lost. However, this assumption may be incorrect. Studies in rodents and humans have reported improvements in the glomerular filtration rate (GFR) and albuminuria after bariatric surgery that are independent of change in weight [2–4]. Bariatric surgery also has weight-independent effects on other parameters like glucose metabolism [5]. One putative mechanism for this involves the rapid post-operative rise in circulating glucagon-like peptide-1 (GLP-1), a hormone secreted by intestinal cells that has several physiologic effects, including enhancement of insulin secretion and increased satiety [6]. GLP-1 has also been observed in some but not all studies to mediate changes in GFR and sodium handling [6–10]. The potent effects of GLP-1 are manifested in the widely used drug class of GLP-1 agonists.

In order to better understand the impact of weight loss on GFR and circulating GLP-1 and their interrelationship, we performed an exploratory study in which we measured these parameters before, immediately after, and many months after Roux-en-Y gastric bypass. Our hypothesis was that there would be a weight independent effect on GFR that would be associated with changes in circulating GLP-1.

## Methods

### Participants

Ten bariatric surgery patients were recruited from the Indianapolis, IN, area with approval of the local investigational review board (#1105005352) and a data safety & monitoring board. Exclusion criteria included pregnancy, iodine allergy, cimetidine or trimethoprim use, dialysis dependency and an expected life span of <2 years. Pregnancy was excluded by urine test. Six participants never completed the protocol due to timing of surgery, illness, or surgical complications. The remaining four patients underwent Roux-en-Y gastric bypass (RYGB). Two of the patients had insulin-dependent diabetes and were instructed to hold their oral diabetes medications and insulin the day of the procedure.

### Measurements

Patients fasted overnight before each visit. GFR was calculated from iohexol (Omnipaque-300; GE Healthcare, Piscataway, NJ) plasma clearance using a six-sample method drawn over 6 h. Blood samples were drawn from a separate intravenous catheter. Plasma was isolated and stored at −80 C° until measured. Plasma (100 µL) underwent protein precipitation with acetonitrile following addition of internal standard para-aminobenzoic acid (PABA). Supernatant was evaporated and reconstituted in 0.1% formic acid, 5% acetonitrile, 95% water and separated using a Phenomenex C18 5uM 4.6 × 150 nm column with gradient to 95% acetonitrile over 15 min on an Agilent 1100 HPLC (Santa Clara, CA) attached to a UV detector (λ = 254nm). Individual iohexol clearances (mL/min) were calculated by standard noncompartmental methods using Phoenix 64 v 8.0 (Certera, Princeton, NJ). Iohexol clearance was calculated as dose divided by the area under the plasma concentration-time curve (AUC) from time zero to infinity.

Immediately prior to the injection of iohexol each patient consumed an oral bolus of 120 mL Ensure^®^ Plus (6.6 g protein, 26 g carbohydrate, 5.6 g fat) as a mixed meal tolerance test. Plasma samples were then drawn at six time points over a 2-h period for measurement of GLP-1. GLP-1active was measured using a commercial ELISA kit (Millipore EZGLPHS-35K). The limit of detection for GLP-1active was 0.14 pM. All samples were run in duplicate on the same assay plate. We previously reported that a mixed meal tolerance test provides a strong stimulus for GLP-1 release [11].

### Statistics

Peak GLP-1 levels after the mixed meal were identified at 20 min. Changes between peak and baseline (“Delta”) were calculated. Given the preliminary nature of this study and the small sample size, we used a one-sample *t*-test to test if the average of differences between two particular time points of interest for each individual (e.g. participant #1, visit 1 vs. visit 2) is equal to zero (SPSS, Armonk, NY). No adjustments were made for multiple tests. Figures were made using SAS (Cary, NC).

## Results

**Figure 1.**
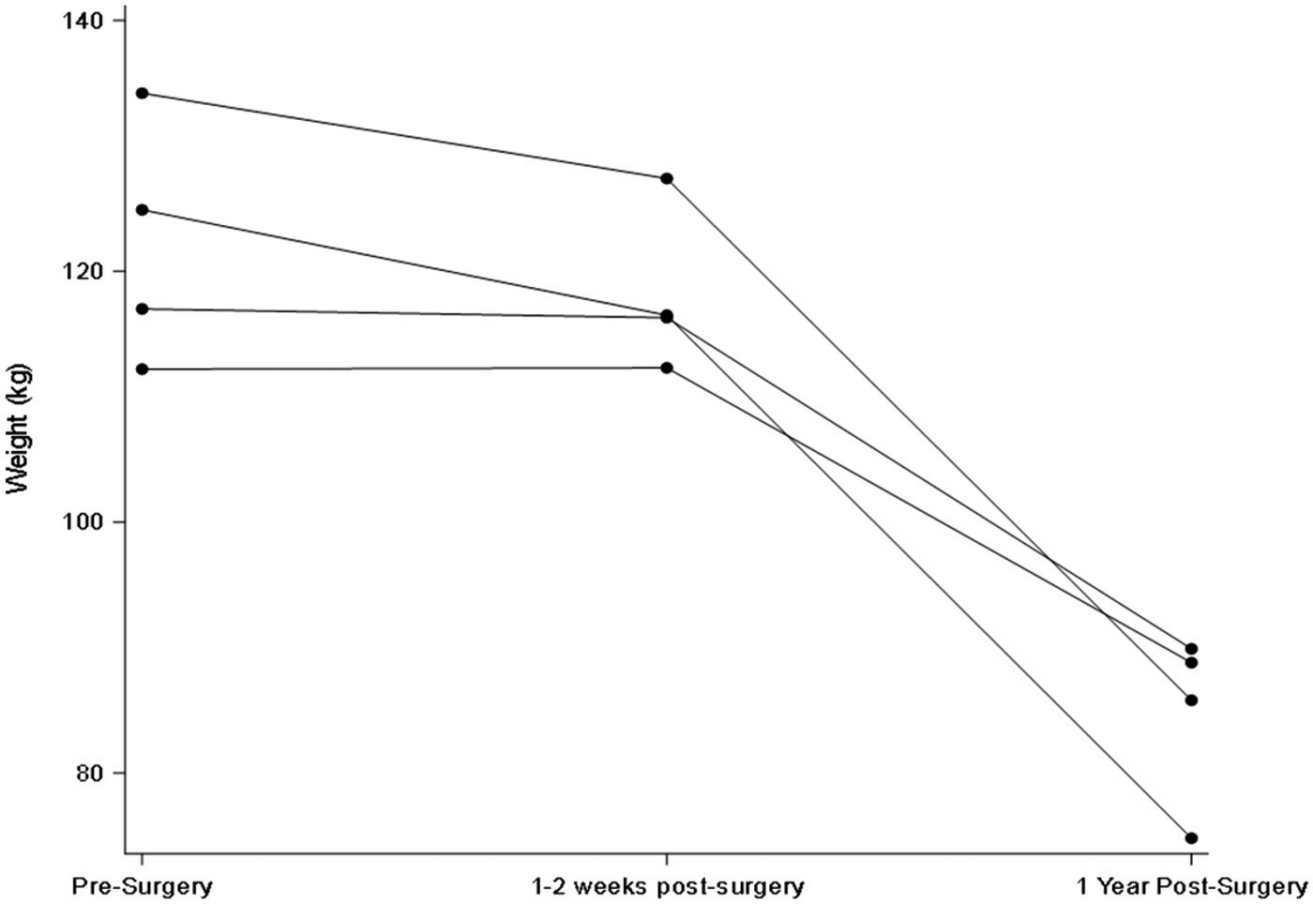
Early and later changes in weight after Roux-en-Y gastric bypass.

**Figure 2.**
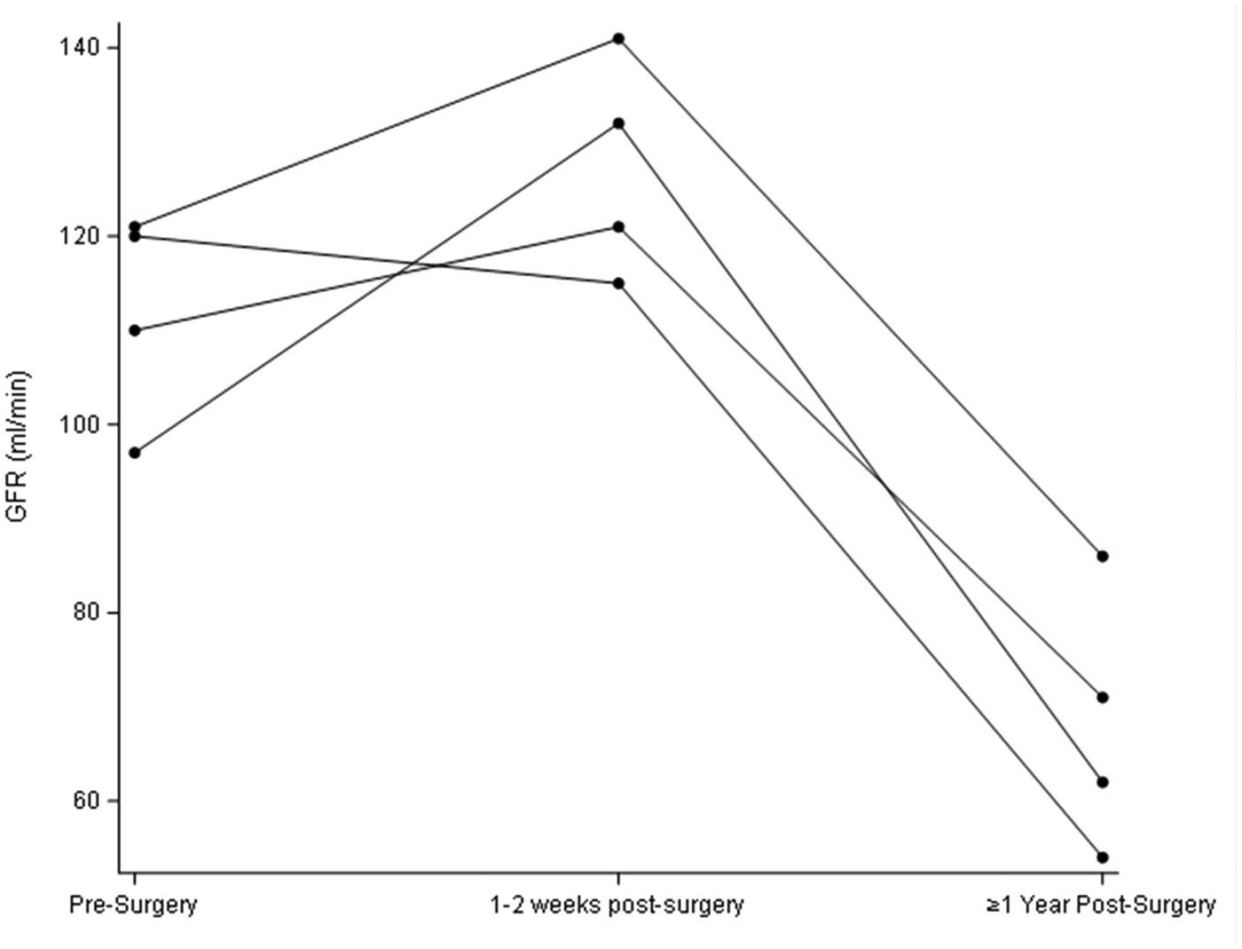
Early and later changes in measured GFR after Roux-en-Y gastric bypass.

**Figure 3.**
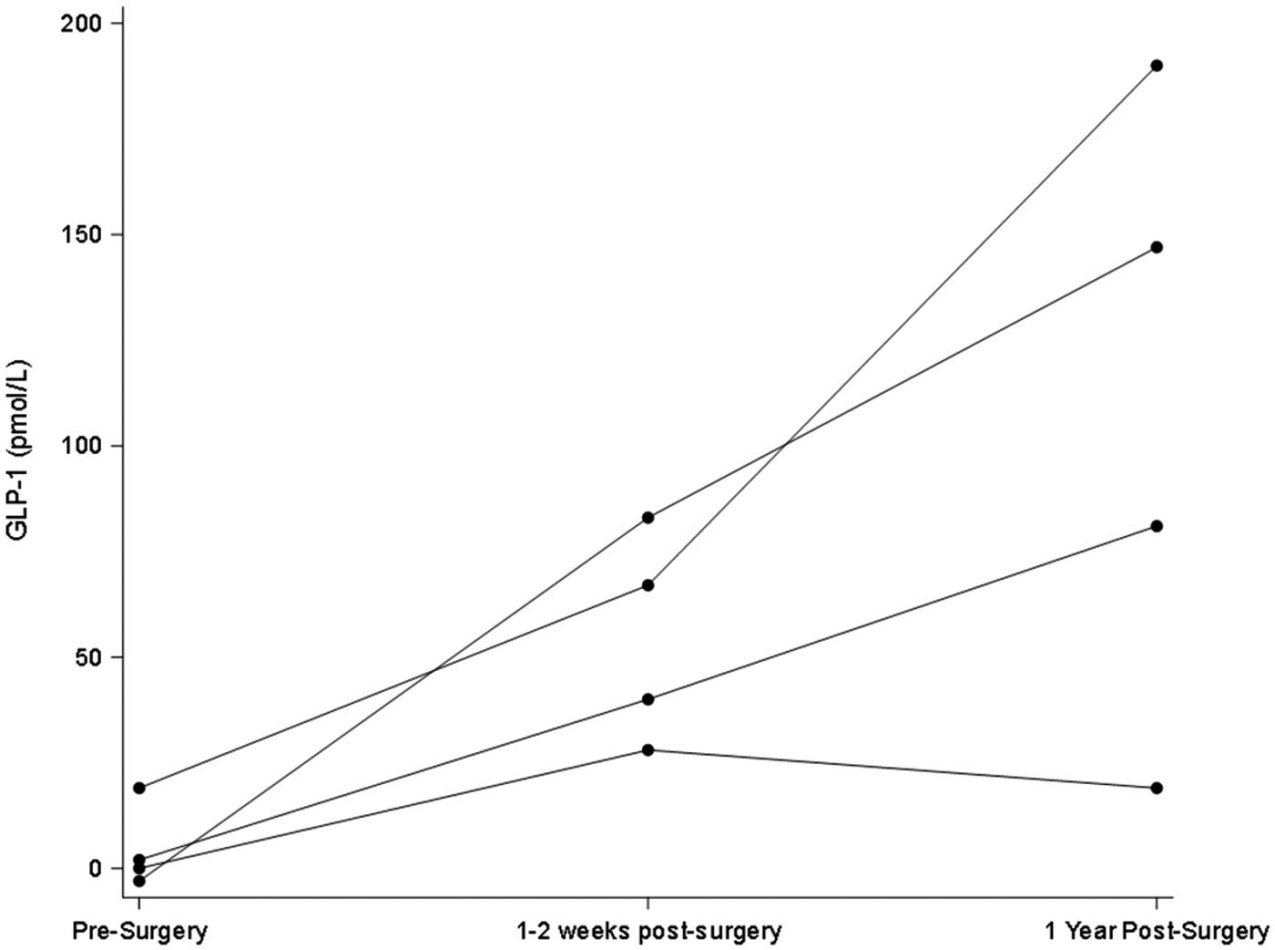
Early and later changes in Delta_20min-baseline_ in GLP-1 after Roux-en-Y gastric bypass. Peak GLP-1 is defined as the difference in levels from baseline to 20 min after a mixed meal.

All participants were female and were 58, 59, 61, and 64 years old. GFR and weight remained statistically unchanged in the first early visit after RYGB as compared to pre-surgery but were both significantly lower in the longer term (Table 1; Figures 1 and 2). In contrast, stimulated GLP-1 was higher at both post-surgery time points compared to baseline (Table 1; Figure 3). Of note, there was no correlation between change in weight and change in GFR (*p* = 0.29) at the early post-operative time point. There was also no clear pattern between the presence of diabetes and changes in GFR and GLP-1.

**Table 1.** Measurements^a^ at each study visit.

Variable	Study visit			*p*-Value**		
	V1 (pre-surgery)	V2 (early post-surgery)	V3 (late post-surgery)	V1 versus V2	V1 versus V3	V2 versus V3
Timing relative to bariatric surgery (days)	−6 to −3	8 to 16	344 to 487	—	—	—
GFR (ml/min)	97 to 121	115 to 141	54 to 86	0.17	0.01	<0.01
Weight (kg)	112.2 to 134.2	112.3 to 127.4	74.8 to 89.9	0.16	0.01	<0.01
% Total weight loss (kg)	—	0 to 6.7	20.9 to 40.1	—	—	<0.01
Fasting basal levels GLP-1 (ρmol/L)	2 to 49	5 to 18	2 to 29	0.83	0.81	0.91
Stimulated levels after mixed meal test GLP-1 (ρmol/L)	−3 to 19	28 to 83	19 to 190	0.03	0.06	0.14

V1: Visit 1; V2: visit 2; V3: visit 3.

^a^Range.

**By one-sample *t*-test. *P*-value for testing whether the difference between different means is greater than zero.

## Discussion

Studies of bariatric surgery associated-weight loss on kidney hemodynamics have traditionally compared pre-surgery GFR levels to levels measured 1 year or later post-surgery. This exploratory study is the first to have directly measured GFR both very early and later after bariatric surgery to determine if changes in GFR are independent of weight loss and correlated with changes in GLP-1. The results offer insight into the gut-kidney axis and the associated impact of bariatric surgery.

While we found, consistent with earlier reports, that the long-term GFR is lower after RYGB compared to preoperative levels [2], we observed no reduction in GFR in the early post-operative period. This is notable because major metabolic improvements in the bariatric milieu are known to occur at that time point even before appreciable weight has been lost [12]. This weight-independent effect is believed to be mediated by rapid changes in circulating hormones and gut peptides such as GLP-1 and possibly others [12]. The lack of a fall in GFR early on after bariatric surgery suggests that the humoral changes occurring at that time do not significantly influence GFR or, alternatively, that countervailing effects are also upregulated. Incidentally, and consistent with our previous report [13], GFR was also not appreciably influenced by the sharp reduction in dietary protein intake that typically occurs immediately after bariatric surgery.

Obesity-associated glomerular hyperfiltration is hypothesized to result from afferent arteriolar vasodilation either directly or indirectly through deactivation of tubuloglomerular feedback [14]. GLP-1 is part of the gut-brain-kidney axis that helps mediate appetite, weight, and metabolism and has also been reported to have variable effects on regulating GFR and renal tubular sodium reabsorption (which itself can influence tubuloglomerular feedback) [6–10]. While the GFR remained unchanged early post-operatively compared to baseline, stimulated GLP-1 secretion was as expected significantly higher (Table 1; Figure 3). Of note, fasting basal GLP-1 levels also did not track well with changes in GFR (data not shown).

While our findings are certainly preliminary, it is tempting to use them to reevaluate our understanding of the determinants of obesity-related glomerular hyperfiltration. A recent study by Chagnac and colleagues concludes that the deactivated tubuloglomerular feedback seen in obese individuals is a result of increased proximal tubular sodium reabsorption, a finding that would be expected to reverse early after weight loss [15] as sodium avidity drops and natriuresis occurs [16]. The lack of a reduction in GFR in our cohort early after bariatric surgery raises the possibility that other mechanisms are contributing to glomerular hyperfiltration. One potential candidate is glomerular hypertrophy, which leads to a higher GFR through increased glomerular capillary surface area. It is possible that glomerular hypertrophy may require longer periods than just a couple of weeks to regress, thus explaining why we did not observe a lower GFR early after bariatric surgery.

Our study was limited by small size and the inclusion of a mixed population (persons with and without diabetes). Despite these drawbacks, we were still able to identify statistically significant differences at several times points between variables without any clear distinction noted between persons with or without diabetes. Additionally, GFR measurements were not performed in the fasting state at each visit as is traditionally done in order to stimulate a GLP-1 response as protocol usually requires. However, the amount of protein we provided (∼0.05 g/kg) to stimulate GLP-1 release was far smaller than the protein stimulus usually used to measure renal reserve (∼1 g/kg). Due to restrictions on available resources, we were also unable to analyze a complete array of candidate hormones and collect urine to assess sodium handling. Such testing should be certainly performed in future studies with larger populations.

In summary, we found no weight-independent effect of Roux-en-Y gastric bypass on GFR nor any association between GFR and circulating GLP-1. These findings, if confirmed in larger studies, will help steer future enquiries in this area.
